# The hemibiotrophic cacao pathogen *Moniliophthora perniciosa* depends on a mitochondrial alternative oxidase for biotrophic development

**DOI:** 10.1111/j.1469-8137.2012.04119.x

**Published:** 2012-06

**Authors:** Daniela P T Thomazella, Paulo José P L Teixeira, Halley C Oliveira, Elzira E Saviani, Johana Rincones, Isabella M Toni, Osvaldo Reis, Odalys Garcia, Lyndel W Meinhardt, Ione Salgado, Gonçalo A G Pereira

**Affiliations:** 1 Laboratório de Genômica e Expressão, Departamento de Genética, Evolução e Bioagentes, Instituto de Biologia, Universidade Estadual de Campinas (UNICAMP)CP 6109, Campinas, SP 13083-970, Brazil; 2 Departamento de Biologia Vegetal, Instituto de Biologia, Universidade Estadual de Campinas (UNICAMP)CP 6109, Campinas, SP 13083-970, Brazil; 3 Sustainable Perennial Crops LaboratoryUSDA–ARS, 10300 Baltimore Ave., Bldg. 001, Beltsville, MD 20705-2350, USA

**Keywords:** alternative oxidase (AOX), cacao (*Theobroma cacao*), hemibiotrophic, *Moniliophthora perniciosa*, nitric oxide (NO), phase transition, witches’ broom disease (WBD)

## Abstract

- The tropical pathogen *Moniliophthora perniciosa* causes witches’ broom disease in cacao. As a hemibiotrophic fungus, it initially colonizes the living host tissues (biotrophic phase), and later grows over the dead plant (necrotrophic phase). Little is known about the mechanisms that promote these distinct fungal phases or mediate the transition between them.
- An alternative oxidase gene *(Mp-aox*) was identified in the *M. perniciosa* genome and its expression was analyzed througout the fungal life cycle. In addition, the effects of inhibitors of the cytochrome-dependent respiratory chain (CRC) and alternative oxidase (AOX) were evaluated on the *in vitro* development of *M. perniciosa*.
- Larger numbers of *Mp-aox* transcripts were observed in the biotrophic hyphae, which accordingly showed elevated sensitivity to AOX inhibitors. More importantly, the inhibition of CRC prevented the transition from the biotrophic to the necrotrophic phase, and the combined use of a CRC and AOX inhibitor completely halted fungal growth.
- On the basis of these results, a novel mechanism is presented in which AOX plays a role in the biotrophic development of *M. perniciosa* and regulates the transition to its necrotrophic stage. Strikingly, this model correlates well with the infection strategy of animal pathogens, particularly *Trypanosoma brucei*, which uses AOX as a strategy for pathogenicity.

## Introduction

Fungal plant pathogens have traditionally been classified on the basis of their feeding mechanism into biotrophic, necrotrophic and hemibiotrophic pathogens. Biotrophs are defined by a dependence on the host to complete their life cycle; they derive nutrients from living host cells by forming specialized infection structures, such as haustoria. By contrast, necrotrophs kill the plant and derive nutrients from the dead host cells. In some pathosystems, the pathogen initially keeps cells alive (biotrophic phase), but then kills them at later stages of infection (necrotrophic phase), which characterizes the hemibiotrophic lifestyle (Glazebrook, 2005). Despite being a critical step for the success of most hemibiotrophic infections, the biotrophic phase is, in general, transient and asymptomatic, whereas the necrotrophic phase causes the main disease symptoms in the plant (Munch *et al.*, 2008). The factors responsible for triggering the switch from biotrophy to necrotrophy in hemibiotrophic fungi are still poorly understood, but may depend on key alterations in the pathogen’s metabolic status (Dufresne *et al.*, 2000; Pellier *et al.*, 2003).

Some of the world’s most devastating phytopathogens display a hemibiotrophic lifestyle. For instance, the hemibiotrophic ascomycete *Magnaporthe oryzae*, which causes the rice blast disease, has emerged as a model for the study of phytopathogenic fungi because of its experimental tractability and economic importance (Dean *et al.*, 2005; Wilson & Talbot, 2009). This pathogen initially colonizes host tissues as a biotroph, without causing detectable symptoms. Approximately 72–96 h after infection, lesions become apparent in the plant, characterizing the necrotrophic growth of *M. oryzae*. No morphological differences appear to exist between these two developmental stages (biotrophic and necrotrophic) in *M. oryzae* (Berruyer *et al.*, 2006). Similarly, the hemibiotrophic lifestyle described for many *Colletotrichum*, *Fusarium* and *Phytophthora* species combines an initial short and asymptomatic period of biotrophic growth that is followed by a destructive necrotrophic phase during which symptoms develop.

The basidiomycete *Moniliophthora perniciosa* (formerly *Crinipellis perniciosa*) causes one of the most devastating diseases of cacao (*Theobroma cacao*), namely witches’ broom disease (WBD). The *M. perniciosa*–cacao interaction is classified as hemibiotrophic (Purdy & Schmidt, 1996); however, it has certain peculiar features that make its study of prime importance in the field of plant pathology. In contrast with other well-documented hemibiotrophic infections, the biotrophic and necrotrophic phases of *M. perniciosa* are morphologically distinct: the first phase is monokaryotic and the second phase is dikaryotic with clamp connections for nuclear transfer (Meinhardt *et al.*, 2008). Moreover, the biotrophic stage is unusually extended, lasting for 2–3 months in infected tissue. In this stage of WBD, only a few fungal cells are detected; however, drastic biochemical and morphological alterations are observed in the plant, such as the formation of modified stems, termed ‘green brooms’ (Scarpari *et al.*, 2005). By contrast, necrotrophic growth occurs only during the final stages of infection, when the fungus rapidly colonizes the dead plant tissue, which is called ‘dry broom’, and produces the fruiting bodies (basidiomata) for reproduction (Purdy & Schmidt, 1996).

In *M. perniciosa*, the factors involved in the switch from biotrophy to necrotrophy seem to be dependent on the availability of soluble nutrients and specific carbon sources (Meinhardt *et al.*, 2006). Attempts to obtain the monokaryotic (biotrophic) mycelium under *ex planta* conditions were initially limited by the rapid transition of this primary fungal stage to the dikaryotic (necrotrophic) phase. The first report of the successful maintenance of the *M. perniciosa* monokaryotic phase under laboratory conditions was in a study of a dual culture of basidiospores with potato callus (Griffith & Hedger, 1994). More recently, the development of a medium with glycerol as the sole carbon source allowed for the extended growth of a biotrophic-like (monokaryotic) mycelium under axenic conditions. However, this mycelial stage is still considerably unstable when grown in culture media (Meinhardt *et al.*, 2006).

In general, laboratory cultivation of the biotrophic stage of fungal species is an uncommon and notable achievement in phytopathology. The vast majority of biotrophic pathogens (*Puccinia* spp., *Melampsora* spp., *Blumeria graminis*) are not cultivable outside of their hosts (Spanu & Kamper, 2010). A counterexample is the maize pathogen *Ustilago maydis*, a well-known cultivable biotroph with a dimorphic lifestyle. In contrast with *M. perniciosa*, the monokaryotic form of *U. maydis* is nonpathogenic and is followed by a dikaryotic parasitic form. Moreover, its monokaryotic phase occurs as yeast-like cells that saprotrophically colonize soil and dead organic matter. In the laboratory, this saprotrophic stage is easily cultivated under axenic conditions and, as in *M. perniciosa*, the *ex planta* culture of the biotrophic hyphae of *U. maydis* is only obtained under specific growth conditions (Day & Anagnostakis, 1971).

Analysis of the *M. perniciosa* genome (Mondego *et al.*, 2008) led to the identification of a gene coding for a mitochondrial alternative oxidase (AOX). This protein is a ubiquinol oxidase that catalyzes the reduction of molecular oxygen to water (Elthon & McIntosh, 1987). It constitutes an alternative respiratory pathway to the ‘conventional’ and universally conserved cytochrome-dependent respiratory chain (CRC), which involves respiratory complexes III and IV. In contrast with cytochrome *c* oxidase (complex IV), electron transfer through AOX does not involve phosphorylation, and the redox energy is released as heat instead of being used for ATP production (Van Aken *et al.*, 2009). Consequently, the use of the alternative pathway results in only one coupling site for oxidative phosphorylation (complex I), and energy production, based on the amount of ATP, is approximately one-third of that generated by the CRC pathway (Siedow & Umbach, 2000).

AOX occurs in all plants, as well as in certain fungi, protists, bacteria and animals (Elthon & McIntosh, 1987; Joseph-Horne *et al.*, 2001; Stenmark & Nordlund, 2003; Chaudhuri *et al.*, 2006; McDonald *et al.*, 2009). Although broadly conserved, the precise role of this enzyme has only been well characterized in thermogenic plants, such as those of the family Araceae. During the reproductive process of these plants, AOX is a central enzyme and generates heat to volatilize amines and attract pollinators (Wagner *et al.*, 2008). For all other organisms, the function of this alternative respiratory route has been the subject of much debate. The most accepted role assigned to this enzyme is its participation in the stress response associated with CRC restriction, which can be provoked by complex III and IV inhibitors, such as antimycin-A, carbon monoxide, cyanide and nitric oxide (NO). In the presence of these compounds, AOX bypasses complexes III and IV and acts as an overflow valve for electron transport, thus preventing the deleterious oxidative stress associated with the increased generation of mitochondrial reactive oxygen species (ROS) (Maxwell *et al.*, 1999; Ruy *et al.*, 2006; Vanlerberghe *et al.*, 2009).

NO is an important component of the immune response in animals and plants. Apart from its role as a plant signaling molecule, it acts as a potent inhibitor of the pathogen CRC pathway (complex IV), leading to the generation of deleterious oxidative stress (Brown, 1999). Studies with the animal pathogens *Histoplasma capsulatum*, *Trypanosoma brucei*, *Aspergillus fumigatus*, *Paracoccidioides brasiliensis* and *Cryptococcus neoformans* have proposed that AOX mitigates the oxidative and/or nitrosative stress induced by the oxidative burst and NO generation during host infection (Akhter *et al.*, 2003; Nittler *et al.*, 2005; Chaudhuri *et al.*, 2006; Magnani *et al.*, 2008; Hernandez *et al.*, 2011). Based on these studies, AOX has also been proposed to be a resistance mechanism of phytopathogens to evade plant defenses and successfully complete the infection process (Joseph-Horne *et al.*, 2001; Albury *et al.*, 2009). However, this hypothesis has not been confirmed.

In this study, we present strong evidence for a biological role of AOX in the life cycle of a plant pathogen. The *M. perniciosa aox* gene was characterized, and a clear correlation between the hemibiotrophic lifestyle of this fungus and the functionality of the mitochondrial respiratory routes was observed. In addition, this work suggests that AOX may enable respiration to continue during the first stages of WBD, when the pathogen needs to survive in a hostile environment established by the host defense system (e.g. NO burst). Finally, we show that the inhibition of CRC maintained the fungus in its monokaryotic stage, which may be sustained by AOX-dependent respiration. These data indicate that mitochondrial respiratory pathways and cellular energetic status may play a role in the phase transition and development of phytopathogens.

## Materials and Methods

### Biological material

The experiments were performed using the *Moniliophthora perniciosa* (Stahel) Aime & Phillips-Mora, (2005) strain FA553 (Mondego *et al.*, 2008). The necrotrophic mycelium was used to produce basidiomata and basidiospores, as described by Griffith & Hedger (1993), with modifications introduced by Niella *et al.* (1999). The monokaryotic (biotrophic-like) mycelium was obtained from basidiospores germinated in a defined medium developed by Meinhardt *et al.* (2006). Transfer of the biotrophic-like mycelium to the nutrient-rich medium MYEA (Malt Yeast Extract Agar) (20 g l^−1^ malt extract, 5 g l^−1^ yeast extract and 20 g l^−1^ agar) induced the transition to the necrotrophic phase. Cultures were maintained at 28°C with agitation of 120 rpm when cultured in liquid media.

*Theobroma cacao* L. variety ‘comum’ (Catongo type) was used for infections. Plants were grown for *c.* 3 months in a glasshouse under controlled temperature (26°C) and humidity (> 80%) and a photoperiod of 12 h. Active apical meristems were inoculated with 30 μl of a basidiospore suspension (10^5^ spores ml^−1^), followed by incubation in a humid chamber for 24 h (Frias *et al.*, 1995).

### Mitochondrial isolation and measurement of oxygen consumption

AOX activity was evaluated by measuring the oxygen consumption profile of mitochondria isolated from necrotrophic hyphae, the mycelial stage from which a sufficient amount of organelles can be extracted. Mitochondria isolation was conducted according to the protocol described by Gredilla *et al.* (2006). Protein concentration was determined using the Bradford method with BSA as a standard (Bradford, 1976). Oxygen consumption was determined at 27°C using a Clark-type electrode connected to an Oxygraph unit (Hansatech, King's Lynn, Norfolk, UK). Aliquots of purified mitochondria were added to the closed reaction chamber containing 1 ml of the standard respiration buffer (300 mM sucrose, 10 mM KH_2_PO_4_, 5 mM MgCl_2_, 1 mM EDTA, 10 mM KCl, 0.1% BSA; pH 7.2). Mitochondria were energized with 10 mM glutamate/malate, followed by the addition of 1 mM ADP. AOX activity was measured in the presence of the CRC inhibitor antimycin-A (10 μM), as the oxygen consumption inhibited by 100 μM *n*-propyl gallate.

### Immunodetection of *M. perniciosa* AOX

Purified mitochondria from *M. perniciosa*, rat (negative control) and *Arabidopsis thaliana* (positive control) were resuspended in 1 × Laemmli buffer (5%β-mercaptoethanol, 3.7% glycerol, 1.1% sodium dodecylsulfate (SDS), 23 mM Tris-HCl pH 6.8, 0.01% bromophenol blue) and heated at 95°C for 5 min. Mitochondrial proteins (12.5 μg) were separated by sodium dodecylsulfate-polyacrylamide gel electrophoresis (SDS-PAGE) and transferred to a polyvinylidene difluoride (PVDF) membrane using standard protocols. The membrane was blocked with 5% nonfat milk in TBST (1 × Tris-buffered saline, 0.1% Tween 20) for 1 h and then incubated with a 1 : 100 dilution of the anti-AOX monoclonal antibody (AOA) (Elthon *et al.*, 1989) in TBST for 16 h. Later, the membrane was incubated with a 1 : 10 000 dilution of the secondary anti-mouse IgG antibody in TBST for 1 h. The signal was detected using the ECL Plus Detection System (GE Healthcare, Little Chalfont, Buckinghamshire, UK) on autoradiography film for 5 min. Subunit 3 of the cytochrome *c* oxidase complex (COX3) was used as endogenous control to demonstrate homogeneity in protein loading of the *M. perniciosa* mitochondrial samples. Three micrograms per milliliter of the anti-yeast COX3 antibody (Invitrogen) were used in the immunoblotting, according to the same procedures as described for the AOA.

### Gene expression assays

The effects of azoxystrobin (75 μM – Syngenta, Basel, Switzerland) and of the NO donor 1-hydroxy-2-oxo-3-(3-aminopropyl)-3-isopropyl-1-triazene (NOC-5; 400 μM – Sigma) on *Mp-aox* gene expression were evaluated by quantitative PCR (qPCR). To this end, necrotrophic hyphae were grown in liquid cultures (2 g l^−1^ malt extract, 5 g l^−1^ yeast extract and 50 ml l^−1^ glycerol) at 28°C and with agitation of 120 rpm. Seven days later, cultures were independently treated with the different compounds for 4 h and then harvested for RNA extraction. For the analysis of *Mp-aox* gene expression along the *M. perniciosa* life cycle, the different fungal stages were obtained as described in the ‘Biological material’ section above. To avoid variations in gene expression associated with different growth conditions, biotrophic and necrotrophic hyphae were grown in the biotrophic-maintaining medium. After 7 d of growth, both mycelia were collected to perform the qPCR assays.

Isolation of total RNA from fungal cells was performed using the Plant RNeasy Minikit (Qiagen), and its concentration and quality were assessed with a NanoDrop ND-1000 spectrophotometer (Wilmington, DE, USA). RNA integrity was evaluated on 1% formaldehyde-agarose gel. Synthesis of cDNA was performed with the Superscript II Reverse Transcriptase kit (Invitrogen) using 1 μg of DNAse I-treated total RNA. The housekeeping gene β-*actin* and the *aox* gene were amplified with the gene-specific primer pairs ACTRTF (5′-CCCTTCTATCGTCGGTCGT-3′) and ACTRTR (5′-AGGATACCACGCTTGGATTG-3′), and AOXRTF (5′-GACGTGCCTTTCGGATAGAG-3′) and AOXRTR (5′-CTTGCCAGGAGGAATGGTT-3′), respectively. All qPCRs were performed in a 16-μl mixture containing 1/16 volume of the cDNA preparation, 1/2 volume of SYBR Green Master Mix (Applied Biosystems, Foster City, CA, USA) and 0.2 μM of each primer. Real-time quantifications were executed using the StepOnePlus system (Applied Biosystems) and the following cycles: 2 min at 50°C, 10 min at 95°C and 40 cycles of 15 s at 95°C, 30 s at 53°C and 60 s at 60°C. Dissociation curves were performed to verify the formation of primer dimers and nonspecific amplifications. Data analysis was completed using the mathematical method proposed by Livak & Schmittgen (2001).

RNA-seq libraries produced as part of the WBD Transcriptome Atlas (P. J. Teixeira, unpublished) were inspected for the expression of the *Mp-aox* gene during the *in planta* development of *M. perniciosa* in green brooms and dry brooms.

### Fluorometric measurement of NO in cacao plants

Apical meristems of healthy plantlets and infected cacao seedlings at the green broom stage were collected to evaluate NO generation using the fluorescent indicator 4,5-diaminofluorescein (DAF-2) (Seligman *et al.*, 2008). Approximately 80 mg of each sample were incubated in 0.1 M phosphate buffer (pH 7.2) containing 25 μM DAF-2 for 1 h under dark conditions at room temperature. After removing the tissue and diluting the resulting solution 2.5-fold, fluorescence emission spectra between 500 and 550 nm were recorded with an excitation of 495 nm using a Hitachi F-2500 spectrofluorometer (Hitachi, Chiyoda, Tokyo, Japan). Data were collected from triplicate samples, and fluorescence was expressed in arbitrary fluorescence units.

### Inhibition of mycelial growth assay

The effects of CRC and AOX inhibitors on the growth of *M. perniciosa* in the biotrophic and necrotrophic phases were tested *ex planta*. Azoxystrobin (2.5 μM – Syngenta) was used as a CRC inhibitor (Affourtit *et al.*, 2000) and salicylhydroxamic acid (SHAM; 5 mM – Sigma) as an inhibitor of the alternative pathway (Tanton *et al.*, 2003). These drugs were added individually or simultaneously to the MYEA standard growth medium. The importance of each mitochondrial pathway (CRC and alternative) was assessed by evaluating the effects of these respiratory inhibitors on the growth of the biotrophic and necrotrophic mycelia. To obtain the biotrophic-like hyphae, we germinated *M. perniciosa* basidiospores in the biotrophic-maintaining medium described by Meinhardt *et al.* (2006). After *c.* 7 d of growth, we transferred the biotrophic mycelium to MYEA medium containing the different inhibitors. The necrotrophic mycelium is highly stable and easily maintained under standard laboratory conditions. Therefore, this fungal stage was directly inoculated in MYEA medium with inhibitors, without the need to preculture in a specific medium. To further confirm the effects of SHAM on *M. perniciosa* development, a second AOX inhibitor (*n*-propyl gallate) was also tested.

## Results

### *Mp-aox* gene expression, protein production and AOX activity are coordinately regulated in *M. perniciosa*

The *aox* gene in *M. perniciosa* (*Mp-aox –* GenBank accession number JF826501) is a single-copy gene of 1616 nucleotides and comprises ten exons and nine introns. *Mp-aox* encodes a protein of 378 amino acids that has the conserved motifs described for other plant and fungal AOXs (Vanlerberghe & McIntosh, 1997; Tanton *et al.*, 2003; Albury *et al.*, 2009; McDonald, 2009). Moreover, no significant differences were found between the primary structure of *M. perniciosa* AOX (Mp-AOX) and those described in other fungi (data not shown).

**Fig. 1:**
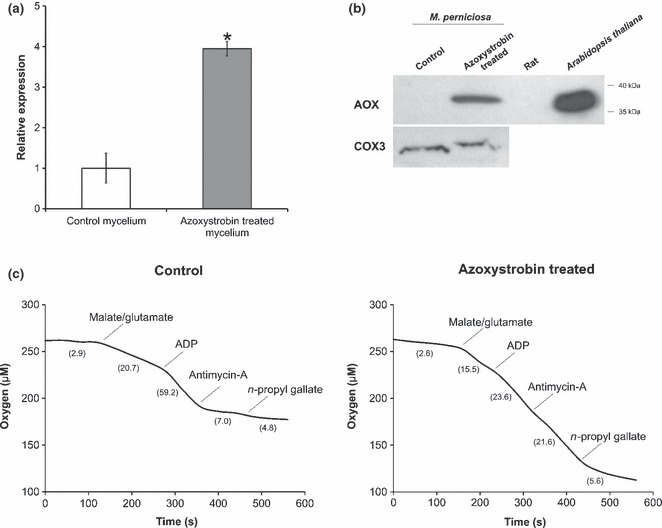
*Mp-aox* gene expression, protein production and alternative oxidase (AOX) activity are well correlated in *Moniliophthora perniciosa*. (a) Relative expression levels of the *Mp-aox* gene in azoxystrobin-treated vs nontreated cells of the necrotrophic hyphae analyzed by quantitative PCR assays. The β-*actin* gene was used as an endogenous control (Student’s *t*-test: *, *P* < 0.01; Error bars represent standard deviation −SD). (b) Immunoblotting assay showing the presence of the Mp-AOX protein in mitochondria isolated from azoxystrobin-treated mycelium of *M. perniciosa*. No signal was detected in mitochondria of nontreated hyphae. The subunit 3 of the cytochrome *c* oxidase complex (COX3) was used as endogenous control. Rat and *Arabidopsis thaliana* mitochondria were used as negative and positive controls, respectively. (c) Oxygen consumption by purified mitochondria from the necrotrophic mycelium of *M. perniciosa.* Mitochondrial suspensions were added to the oxygraph chamber containing the reaction medium and were energized with 10 mM malate/glutamate and 1 mM ADP. AOX activity was assessed in the presence of antimycin-A (10 μM), as the remaining respiration inhibited by *n*-propyl gallate (100 μM). Mitochondrial oxygen consumption of nontreated hyphae showed high sensitivity to antimycin-A, but was unaffected by *n*-propyl gallate. In contrast, mitochondrial oxygen consumption of azoxystrobin-treated cells was poorly affected by antimycin-A, but greatly inhibited by *n*-propyl gallate. The numbers in parentheses represent the oxygen consumption rates in nmol min^−1^ mg^−1^ protein. In all of these assays, azoxystrobin-treated cells were incubated with 75 μM azoxystrobin for 4 h. Control refers to nontreated mycelium.

Gene expression and western blot analyses showed that treatment of the *M. perniciosa* necrotrophic mycelium with the CRC inhibitor azoxystrobin induces *Mp-aox* transcription (Fig. 1a) and increases Mp-AOX protein levels (Fig. 1b), as observed in other plant and fungal species (Minagawa *et al.*, 1992; Sakajo *et al.*, 1993; Vanlerberghe & McIntosh, 1994; Yukioka *et al.*, 1998). In agreement, oxygen consumption by isolated mitochondria of the azoxystrobin-treated mycelium was moderately affected by antimycin-A (CRC inhibitor) and highly sensitive to *n*-propyl gallate (AOX inhibitor) (Fig. 1c). Conversely, the addition of antimycin-A to mitochondrial preparations of nontreated control cells was sufficient to inhibit oxygen consumption (Fig. 1c), showing that the *M. perniciosa* AOX is functional and activated under specific conditions (e.g. CRC inhibition). Moreover, these data suggest that the enzyme is coordinately regulated at gene expression, protein production and activity levels.

### *Mp-aox* expression is up-regulated in *M. perniciosa* during the biotrophic phase

**Fig. 2:**
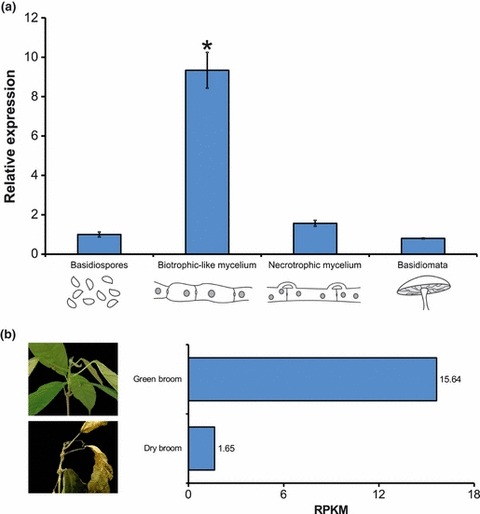
Expression analysis of *Mp-aox* in the *ex planta* and *in planta* development of *Moniliophthora perniciosa*. (a) Relative expression of *Mp-aox* in each developmental stage of *M. perniciosa*– basidiospores, biotrophic-like (monokaryotic) mycelium, necrotrophic (dikaryotic) mycelium and basidiomata – as analyzed by quantitative PCR. β-*actin* gene expression was used as an endogenous control (Student’s *t*-test: *, *P* < 0.01). Representative drawings of each of the life cycle stages are shown. (b) *In planta* expression of *Mp-aox* was assessed after inspection of the witches’ broom disease (WBD) RNA-seq Transcriptome Atlas (P. J. Teixeira, unpublished). Gene expression values are expressed in RPKM (reads per kilobase of exon per million mapped reads).

We evaluated the *ex planta* expression profile of the *Mp-aox* gene to identify specific conditions during the development of *M. perniciosa* in which *Mp-aox* is activated. As shown in Fig. 2(a), gene expression was not significantly different among basidiospores, necrotrophic mycelium and mushrooms (basidiomata). However, a larger amount of *aox* transcripts was verified in the monokaryotic biotrophic-like mycelium when compared with other fungal stages (Fig. 2a). In accordance, measurement of the respiratory rates of these different life stages demonstrated that the biotrophic-like mycelium was the sole fungal stage affected by the AOX inhibitor *n*-propyl gallate (Supporting Information Fig. S1, Methods S1). Finally, supporting these results, inspection of the RNA-seq database (P. J. Teixeira, unpublished) demonstrated that the *M. perniciosa aox* gene is over-expressed in green brooms, when compared with dry brooms, showing a direct correlation between the *ex planta* and *in planta* analyses (Fig. 2b).

### Evaluation of NO generation during the green broom stage of WBD

**Fig. 3:**
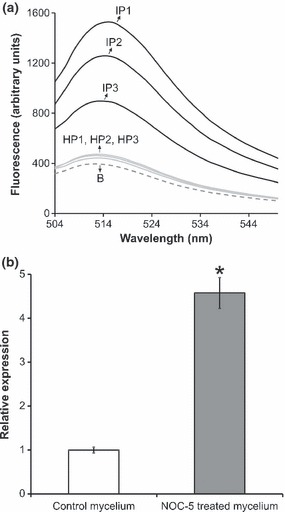
Participation of nitric oxide (NO) in the *Moniliophthora perniciosa*–cacao interaction. (a) Measurement of NO generation by healthy (control) and infected cacao seedlings using the 4,5-diaminofluorescein (DAF-2) fluorimetric method. NO fluorescence of the meristems from each individual plant is shown. The emission at 515 nm corresponds to the maximum fluorescence emitted by DAF-2T, a fluorescent product directly related to the amount of NO in the medium. IP, infected plants; HP, healthy plants; B, blank, which corresponds to the DAF-2 fluorophore diluted in phosphate buffer (0.1 M, pH 7.2) in the absence of tissues. (b) Quantitative PCR assays were performed to evaluate the relative expression of *Mp-aox* in response to the treatment of the necrotrophic hyphae with the NO donor 1-hydroxy-2-oxo-3-(3-aminopropyl)-3-isopropyl-1-triazene (NOC-5) (400 μM) for 4 h (Student’s *t*-test: *, *P* < 0.01).

At *c.* 15 d after inoculation of basidiospores in cacao seedlings, the first symptoms of WBD were observed in the infected plants. Mature green brooms at 22 d post-inoculation were used to evaluate NO generation using the DAF-2 method. Increased amounts of NO were detected in mature green brooms in comparison with healthy, noninoculated plants of the same age (Fig. 3a).

Given that *Mp-aox* expression is elevated in the biotrophic hyphae, we used the necrotrophic mycelium to determine whether this gene is responsive to the radical NO. Following treatment with the NO donor NOC-5, a fourfold induction of *Mp-aox* expression was observed (Fig. 3b). Similar to azoxystrobin, NO potentially inhibits the CRC pathway (Brown, 1999), and *Mp-aox* induction may be a consequence of this process.

### Effects of respiratory inhibitors on the development of the biotrophic-like and necrotrophic mycelia in *M. perniciosa*

**Fig. 4:**
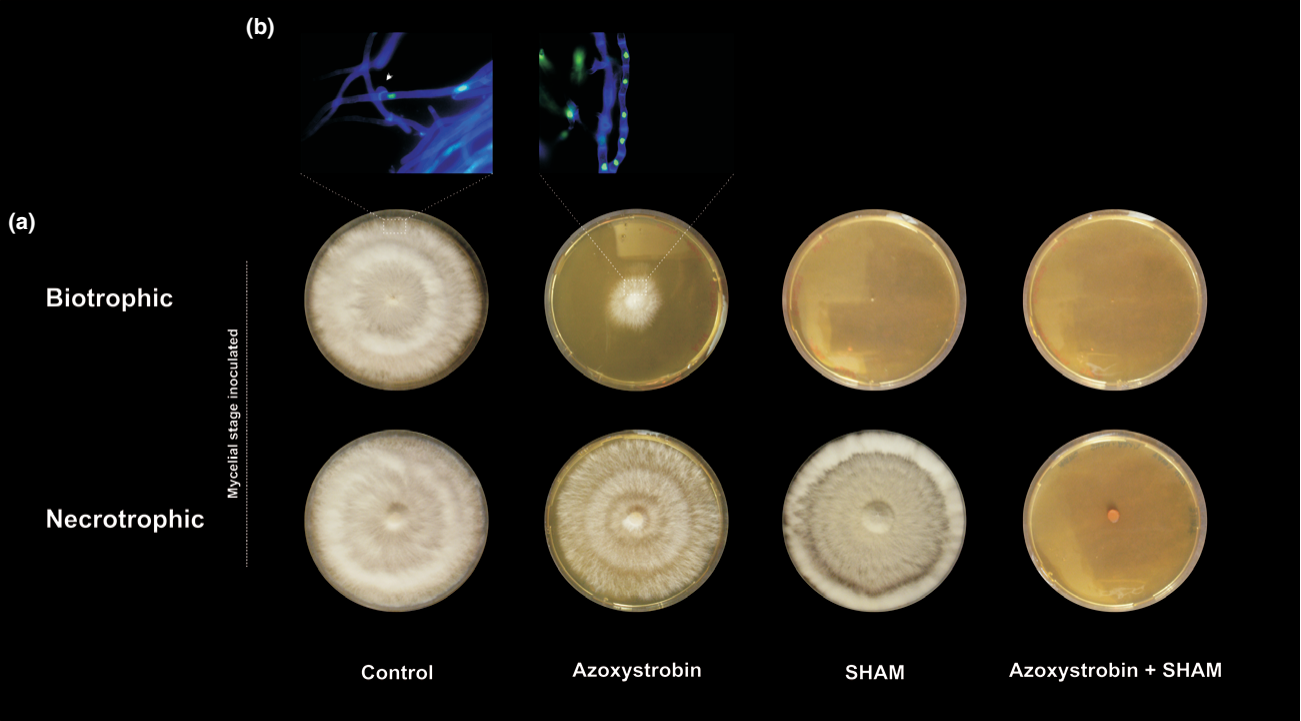
Growth of *Moniliophthora perniciosa* in the presence of the respiratory inhibitors azoxystrobin and salicylhydroxamic acid (SHAM). (a) Monokaryotic biotrophic-like phase (top panel) and dikaryotic necrotrophic phase (bottom panel) were inoculated in culture medium containing the cytochrome-dependent respiratory chain (CRC) inhibitor azoxystrobin (2.5 μM), the alternative oxidase (AOX) inhibitor SHAM (5 mM) or both drugs. Control, no treatment. (b) Microscopy images show that, as expected, in the control, the biotrophic inoculum switched to the necrotrophic phase (the white arrow shows the presence of clamp connections, which are an exclusive feature of the necrotrophic hyphae). By contrast, in the presence of azoxystrobin, the transition was prevented, and the fungus maintained the biotrophic morphology, as indicated by the presence of a single nucleus per cell. All photographs were taken at 21 d post-inoculation.

Because gene-specific mutants of *M. perniciosa* are unavailable, we employed specific inhibitors of the cytochrome and alternative routes of electron transfer to confirm the role of AOX in the metabolism/development of *M. perniciosa*. Azoxystrobin and SHAM were used to inhibit the CRC and alternative pathways, respectively. These drugs were added individually as well as simultaneously to growth medium, subsequently inoculated with either the biotrophic-like or necrotrophic mycelium (Fig. 4).

As reported previously (Meinhardt *et al.*, 2006), inoculation of the biotrophic-like mycelium in standard growth medium caused biotrophic-like hyphae to switch to the necrotrophic phase (Fig. 4a, top panel, Fig. 4b). Analysis of mycelial morphology showed that the switch started between 24 and 48 h after inoculation and, within 5 d, monokaryotic hyphae were no longer observed. Remarkably, in the presence of azoxystrobin (Fig. 4a, top panel, Fig. 4b), the fungus was able to grow for an extended time as a monokaryotic mycelium (*c.* 30 d), suggesting that CRC inhibition, which results in a low energetic status and possibly high AOX respiration, may favor biotrophic development. Furthermore, in the presence of SHAM (Fig. 4a, top panel), biotrophic growth was completely halted, reinforcing the idea that the alternative respiratory route is the primary mitochondrial pathway during this mycelial stage. In addition, we used another AOX inhibitor (*n*-propyl gallate) to further confirm the effects of AOX inhibition on fungal development. As expected, the biotrophic mycelium showed a higher sensitivity to this inhibitor in comparison with the necrotrophic mycelium (Fig. S2).

The necrotrophic mycelium developed in the presence of both respiratory inhibitors – azoxystrobin and SHAM – at the concentrations tested (Fig. 4a, bottom panel). Only when azoxystrobin was combined with the AOX inhibitor SHAM did necrotrophic mycelium development stop completely (Fig. 4a, bottom panel). Therefore, these data indicate that an interplay between the two respiratory pathways (CRC and alternative routes) may exist during this second fungal stage, as confirmed by the results presented in Fig. 1.

## Discussion

*Moniliophthora perniciosa* displays a hemibiotrophic lifestyle with a peculiar mode of nutrition: after initial penetration, it grows for *c.* 60–90 d as a biotroph in the intercellular spaces of the plant – a period of drastic morphological and physiological alterations of the infected tissues (Scarpari *et al.*, 2005). The necrotrophic mycelium appears only during the final stages of the disease, colonizing the dead plant tissues (Meinhardt *et al.*, 2008). The switch between these two growth phases is associated with remarkable morphological, genetic and physiological changes in the hyphae (Meinhardt *et al.*, 2006; Rincones *et al.*, 2008). This study demonstrated that these two life strategies (biotrophic and necrotrophic) use different electron transfer pathways for cell respiration. Although the necrotrophic phase mainly depends on the CRC pathway with an inducible alternative respiration, the biotrophic stage primarily relies on the activity of an alternative respiratory pathway.

Activation of the mitochondrial alternative pathway is associated with a considerable reduction in cell energy production (Elthon & McIntosh, 1987). For this reason, the up-regulation of *Mp-aox* gene expression (Fig. 2a) may explain the slow growth observed during biotrophic development (Meinhardt *et al.*, 2006). Previous work by Affourtit *et al.* (1999) demonstrated that functional expression of the plant *aox* gene in the yeast *Schizosaccharomyces pombe*, which naturally lacks the alternative pathway, significantly reduces total biomass and growth rate, a phenotype that resembles the biotrophic phase in *M. perniciosa* (Meinhardt *et al.*, 2006, 2008). Moreover, in agreement with the dependence of the biotrophic hyphae on AOX respiration, we verified that SHAM and *n*-propyl gallate completely halted biotrophic development, suggesting that AOX inhibition may represent an effective strategy for stalling the progression of WBD during its early stages (Figs 4, S2). A similar strategy has been successfully employed to control sleeping sickness caused by the protozoan *T. brucei*. The infective phase (bloodstream form) of *T. brucei* greatly depends on the activity of the alternative route, and treatment with AOX inhibitors (SHAM/ascofuranone) results in trypanocidal effects (Chaudhuri *et al.*, 2006).

The increased expression of the *Mp-aox* gene (Fig. 2a) and the greater activity of *M. perniciosa* AOX (Fig. S1, Methods S1) in the biotrophic-like mycelium suggest that the enzyme may play a role during this specific fungal stage. Accordingly, inspection of the WBD Transcriptome Atlas (P. J. Teixeira, unpublished) showed that this gene is up-regulated during the green broom stage of WBD (wherein the fungus grows biotrophically) when compared with the dry broom stage (which is colonized by the necrotrophic hyphae) (Fig. 2b). Despite having a negative effect on the fungal growth rate, AOX may be associated with the detoxification of mitochondrial ROS generated during the initial stages of infection (Scarpari *et al.*, 2005; Ceita *et al.*, 2007; Rincones *et al.*, 2008).

In general, plants produce large amounts of NO in response to biotrophic and hemibiotrophic pathogen infection (Delledonne *et al.*, 1998). In addition to activating defense signaling pathways of the plant, NO can inhibit fungal cells directly by disrupting the mitochondrial complex IV, which increases ROS production, causing oxidative and nitrosative stresses (Brown & Borutaite, 2002; Romero-Puertas *et al.*, 2004). Moreover, the NO superoxide derivative peroxynitrite causes damage to a variety of mitochondrial components through oxidizing reactions, leading to the irreversible inhibition of mitochondrial respiration (Brown, 1999; Brown & Borutaite, 2002). In this study, we observed elevated amounts of NO in infected seedlings compared with the levels found in healthy cacao plants (Fig. 3a). Therefore, we speculate that the ability of the biotrophic mycelium to survive in cacao tissues may strongly depend on mechanisms that circumvent NO-induced CRC impairment. In this regard, AOX may provide *M. perniciosa* with protection against the harmful effects of NO and its derivatives on the mitochondrial respiratory chain. In support of this hypothesis, we verified that *Mp-aox* gene expression is up-regulated by the NO donor NOC-5 *ex planta* (Fig. 3b). Thus, our data indicate that increased NO production occurs during host infection, and fungal AOX may play a protective role in overcoming the harmful effects of this compound on the mitochondrial respiratory chain. If so, during biotrophic development, *M. perniciosa* may slow its growth rate in order to focus on detoxification.

In addition to the protective role of AOX against plant defenses, we also suggest that the enzyme may participate in the regulation of the hemibiotrophic development of *M. perniciosa*. We demonstrate that the switch from the biotrophic-like to the necrotrophic phase is prevented by azoxystrobin, and the fungus continues to grow as a biotrophic mycelium for an extended time in the presence of this inhibitor (Fig. 4). Modulation of the switch between biotrophy and necrotrophy in hemibiotrophic fungi has not been well characterized; however, a few studies have demonstrated that this transition depends on key alterations in the pathogen's metabolism (Dufresne *et al.*, 2000; Pellier *et al.*, 2003). Therefore, we propose that the low-energy metabolic status caused by the inhibition of the CRC pathway, together with high AOX respiration, may be important requirements for the maintenance of biotrophic growth, as well as for the control of the phase transition process in *M. perniciosa*.

Previous work by Evans (1980) has suggested that an unstable molecule produced by the plant is responsible for the maintenance of the biotrophic phase in *M. perniciosa*. In the absence of this molecule, the fungus would rapidly switch to the necrotrophic phase. According to our results, *ex planta* maintenance of the biotrophic mycelium is directly related to inhibition of the CRC route. Thus, we propose that plant NO generation during infection may be the factor responsible for the control of biotrophic development *in planta*. We then speculated that, on the death of the plant tissues, NO-dependent inhibition of the respiratory chain would cease and an increase in the cytochrome/AOX ratio would supply the necessary energy (ATP) for intense pathogen growth during the necrotrophic phase. In this regard, AOX may be a switch regulating the development of these two life strategies in *M. perniciosa* (biotrophic and necrotrophic phases). Our study is the first report of the participation of AOX in the development of a plant pathogen, which sheds light on the physiological functions of this enzyme as well as on the mechanisms that control the phase transition process in phytopathogens, a central step during infection.

Finally, we verified that AOX conferred resistance to the fungicide azoxystrobin during both mycelial stages in *M. perniciosa* (Figs 1, 4). This is because the biotrophic-like and necrotrophic hyphae were able to grow in the presence of this CRC inhibitor, but the combination of the two respiratory inhibitors (azoxystrobin and SHAM) was lethal and completely inhibited the development of these mycelia. Indeed, for the majority of other phytopathogens, such as *Mycosphaerella graminicola*, *Botrytis cinerea* and *M. oryzae*, AOX has been shown to function as an effective mechanism to rescue the fungus from oxidative stress conditions induced by azoxystrobin fungicides (Avila-Adame & Koller, 2002; Miguez *et al.*, 2004; Ishii *et al.*, 2009).

Despite the well-documented function of AOX in fungicide resistance, the nature of the environmental stress conditions in which this enzyme plays a role during the development of phytopathogens is still unknown. A primary approach to understand AOX function during the development of a plant pathogen was performed using the hemibiotrophic fungal model *M. oryzae*. However, deletion of the *aox* gene in this fungus had no effect on fungal pathogenicity or life cycle, thus refuting the hypothesis that AOX contributes to the virulence or development of this phytopathogen (Avila-Adame & Koller, 2002).

Although *M. oryzae* is a good and well-characterized model for understanding many hemibiotrophic infections, a remarkable difference exists between the life strategies of *M. oryzae* and *M. perniciosa*. Although the biotrophic phase in *M. oryzae* lasts only a few days (Ribot *et al.*, 2008), *M. perniciosa* exhibits an unusually extended biotrophic stage (60–90 d) during which the fungus is in continuous contact with the living plant tissues (Evans, 1980; Meinhardt *et al.*, 2008; Mondego *et al.*, 2008). During this extended biotrophic phase, the alternative pathway may play a central role in the survival of *M. perniciosa*, which has to deal with a hostile environment established during infection. In this regard, the *M. perniciosa*–cacao pathosystem has more in common with the long-term interactions established between animal pathogens and their hosts (e.g. sleeping sickness caused by *T. brucei*, cryptococcal disease caused by *C. neoformans*, aspergillosis caused by *A. fumigatus*, paracoccidioidomycosis caused by *P. brasiliensis*, etc.) than those described for other hemibiotrophic phytopathogenic fungi (Akhter *et al.*, 2003; Chaudhuri *et al.*, 2006; Magnani *et al.*, 2008; Hernandez *et al.*, 2011). Indeed, a parallel can be drawn between the life strategies of *M. perniciosa* and *T. brucei*. As in *M. perniciosa*, the protozoan *T. brucei* uses an AOX-dependent respiratory pathway during its infective stage inside the mammalian host. However, during its reproductive cycle inside the insect vector, the protozoan’s respiration is primarily dependent on the activity of the cytochrome pathway (Chaudhuri *et al.*, 2006). This strategy seems to help *T. brucei* overcome host defense mechanisms that are mainly based on the induction of oxidative stress (Fang & Beattie, 2003).

In conclusion, this work presents a novel mechanism describing the development of phytopathogens that has a remarkable parallel to the mechanisms evolved in animal parasites. In addition, we believe that our work contributes to the understanding of the alternative respiratory pathway, which appears to be involved in the hemibiotrophic life cycle of a phytopathogenic fungus. Finally, based on the importance of AOX in the *M. perniciosa* life cycle, we believe that this enzyme is a promising target for the development of new chemicals to disrupt *M. perniciosa* development and to effectively control WBD. Whether the enzyme also plays a role in the development of other phytopathogens, particularly in other hemibiotrophic fungi, should be the subject of future studies.

## Supporting Information

**Fig. S1** Measurement of whole cellrespiratory rates of the main life stages of *Moniliophthoraperniciosa*.

**Fig. S2** Effects of *n*-propyl gallateon the *ex planta* development of *Moniliophthoraperniciosa*.

**Methods S1** Measurement of oxygenconsumption by the main life stages of *Moniliophthoraperniciosa.*
