# Supplementary material for: The hemibiotrophic cacao pathogen Moniliophthora perniciosa depends on a mitochondrial alternative oxidase for biotrophic development
Source: New Phytol. 2012 Jun;194(4):1025–34. doi: 10.1111/j.1469-8137.2012.04119.x (PMC3415677; doi:10.1111/j.1469-8137.2012.04119.x)
Supplement: Supplementary file 1 [file nph0194-1025-SD1.pdf]

<u>**Supporting Information Figs S1, S2 and Methods S1**</u>

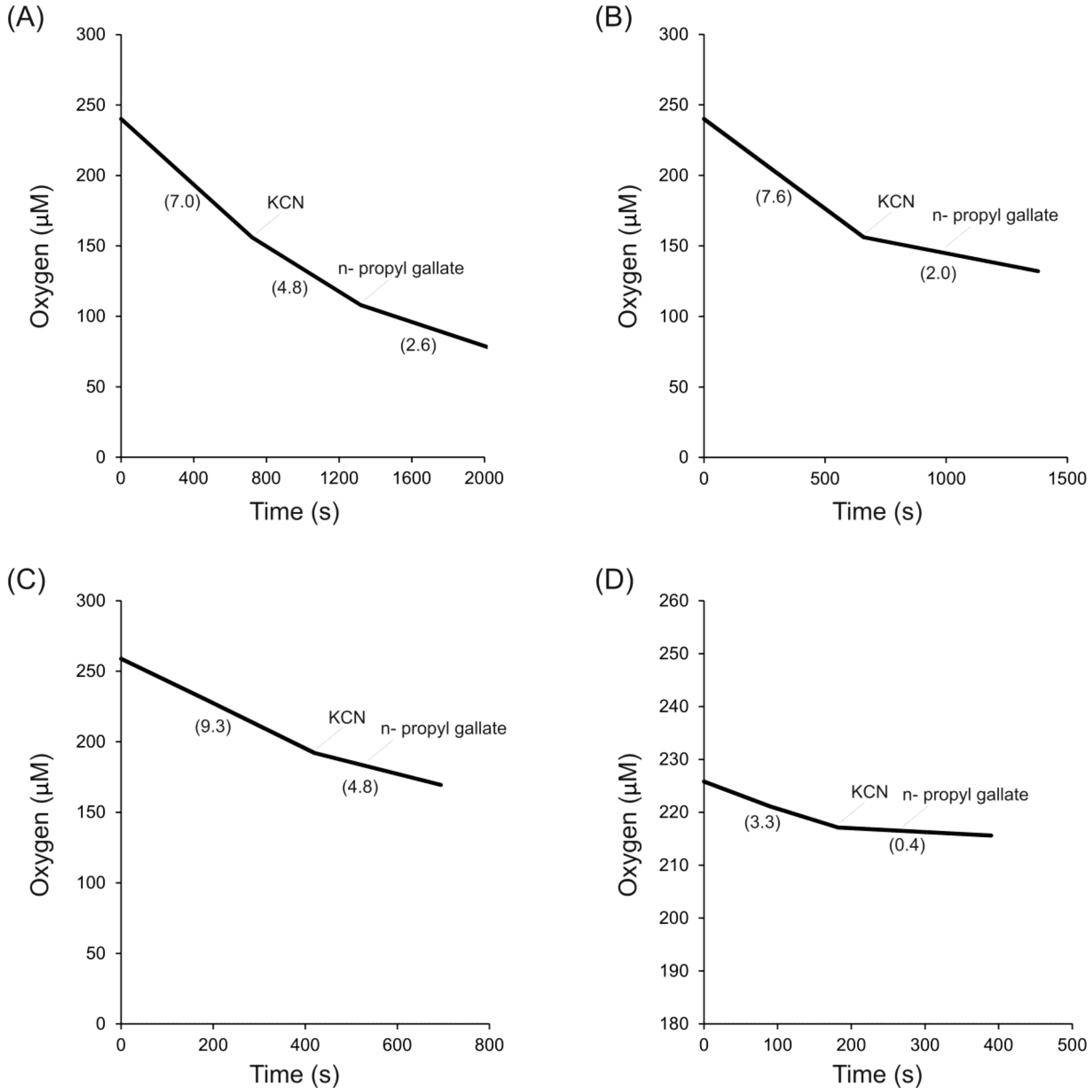

Fig. S1 Measurement of whole cell respiratory rates of the main life stages of *Moniliophthora perniciosa*. (A) Biotrophic-like mycelium, (B) necrotrophic mycelium, (C) basidiomata and (D) basidiospores. Oxygen consumption was evaluated in response to 1 mM potassium cyanide (KCN, a CRC inhibitor), followed by addition of 200 µM *n*-propyl gallate (AOX inhibitor). Oxygen consumption by the biotrophic-like mycelium was the sole condition affected by *n*-propyl gallate, thus confirming that the alternative respiratory chain is active during this developmental stage of *M. perniciosa*.

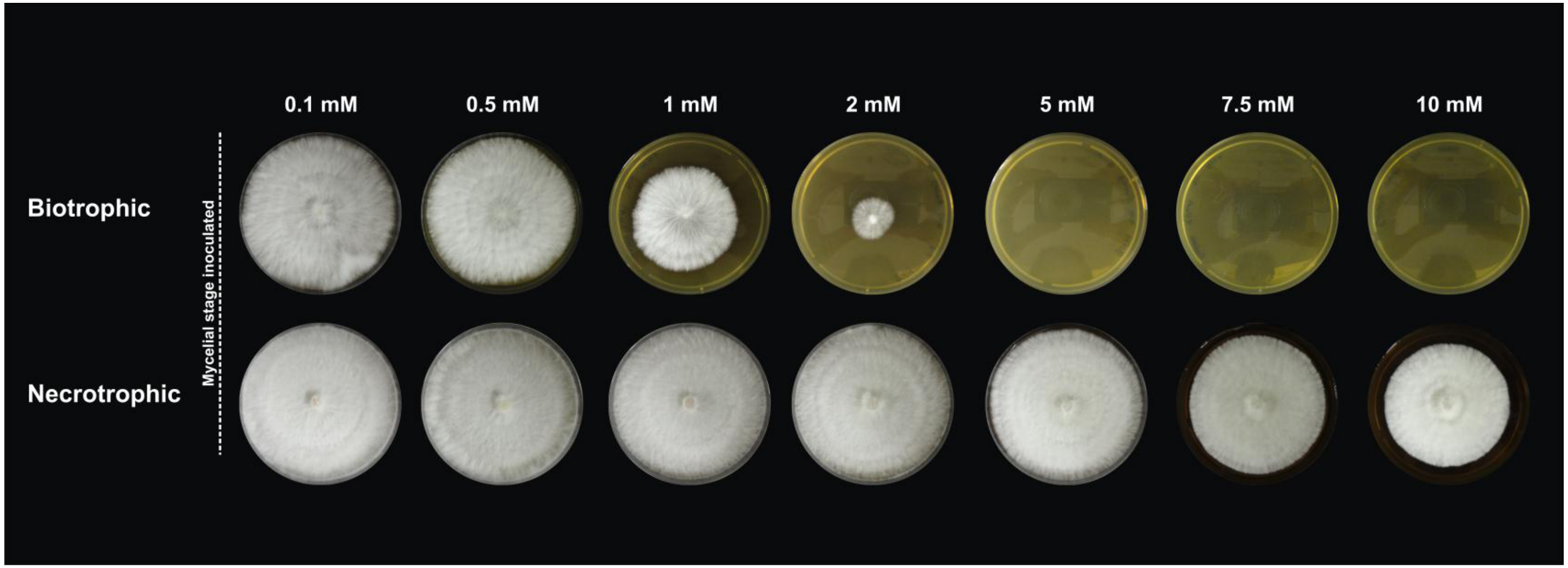

Fig. S2 Effects of *n*-propyl gallate on the *ex planta* development of *Moniliophthora perniciosa*. Monokaryotic biotrophic-like phase (upper panel) and dikaryotic necrotrophic phase (lower panel) were inoculated in culture media containing the AOX inhibitor *n*-propyl gallate in a range of different concentrations (from 0.1 to 10 mM). The biotrophic-like mycelium shows a higher sensitivity to *n*-propyl gallate in relation to the necrotrophic mycelium, further confirming the role of AOX in the development of the monokaryotic hyphae of *M. perniciosa*.

Methods S1 Measurement of oxygen consumption by the main life stages of *Moniliophthora perniciosa.*

We evaluated the oxygen consumption profile by whole cells of the different developmental stages of *M. perniciosa* (basidiospores, basidiomata, biotrophic and necrotrophic mycelia). Oxygen consumption was determined at 27°C using a Clark-type electrode connected to an Oxygraph unit (Hansatech). The experiments were performed using 2.5×10^6 spores, the whole mushroom structure (weighing about 55 mg), and approximately 60 mg of each fungal mycelium (biotrophic and necrotrophic mycelia). These fungal structures were individually added to the closed reaction chamber containing 1 mL of culture medium (2 g l^{-1} malt extract, 5 g l^{-1} yeast extract, and 50 ml l^{-1} glycerol). AOX activity was measured in the presence of the CRC inhibitor potassium cyanide (1 mM) as the oxygen consumption inhibited by 200 µM *n*-propyl gallate.
